# Bibliography

**Published:** 1898-08

**Authors:** 


### Bibliography.

A Text-Book of Dental Pathology and Therapeutics, Including Pharmacology. Being a Treatise on the Principles and Practice of Dental Medicine. For Students and Practitioners. By Henry H. Burchard, M.D., D.D.S. Special lecturer on Dental Pathology and Therapeutics in the Philadelphia Dental College. Illustrated with three hundred and eighty-eight engravings and two colored plates. Philadelphia and New York: Lea, Brothers & Co., 1898.

The past two years has been a prolific period in new dental books, and the present volume, by Dr. Burchard, is a fitting conclusion of his labors on the two preceding books issued by Lea, Brothers & Co.; certainly an extraordinary production from a single house in one line of work.

To the average writer the labor performed by the author upon the previous volumes, as one of the collaborators in each, would seem to have been sufficient for the time being; but this volume of five hundred and eighty-seven pages, including index, is a full attestation to his energy and comprehensiveness of thought in his chosen work.

The author says in his preface, "This volume is designed as a text-book of the principles and practice of dental medicine for the use of students, and as a reference work on applied special pathology and therapeutics for the use of dentists."

The book opens with a chapter on "General Principles," which, it is needless to say, is ably, though somewhat briefly handled by the author. The succeeding two chapters on "Causes of Disease, General and Local Bacteriology, with Special Reference to Dental Pathology and Therapeutics," cover much valuable matter, and are treated with the attention and care due these important subjects.

The fourth chapter, on "Disturbances of Nutrition: Atrophy, Degeneration, Necrosis, Hypertrophy, Tumors," may be regarded as a too limited treatment of those extensive subjects; but it must be remembered in examining a book largely intended for students that brevity is essential. It is doubtful, however, whether the author's subdivision of necrosis is sufficiently full for that very class. This appears to be the only allusion made to this important pathological condition, and is unsatisfactory, for beyond the general principles govering its etiology and progress, nothing is given. It is probable that the author considered this as properly belonging to oral surgery, and hence not appropriate to a work on dental pathology. This is true in part only; in fact, surgery has but a limited part in its treatment. The dental practitioner of the present period must accept all cases of this character, and in his care they should be kept until the time arrives for the surgeon to perform his part. It would seem, therefore, a mistake not to have embodied in this work detailed methods of diagnosis, positive and differential, and treatment during the waiting period, antedating the formation of the sequestrum.

The next chapter, on "Disturbances of the Vascular System," covers, in part, inflammation.

This is treated with the author's usual clearness of expression, but does not, in the view of the writer, meet the demand of those familiar with the steps taken to reach the present intelligent conception of the subject. It would not have added materially to the size of the volume to have made the readers acquainted with the work of Döllinger (1819), Müller (1824), Kaltenbrunner (1826), and Waller; Addison and Williams's work, in England, from 1846 to 1849; also von Recklinghausen, and Beale, upon the character of the white blood-corpuscle, investigations which changed entirely the previous conceptions entertained in regard to this element in the blood, and prepared the minds of investigators for the Waller-Cohnheim theory of inflammation. More allusion, it would seem, should have been made to the older proliferation theory of Virchow and to the work of Stricker and his school in connection with it. The work of Waller, of England, antedating by many years that of Cohnheim, should never be allowed to sink into oblivion and be forgotten in the discussion of the several changes following irritation.

The chapters from I. to VIII., covering one hundred and seventy-nine pages, are, as a whole, worthy of special commendation. They are thoroughly illustrated with the care taken by the author in previous works.

The chapter on dentition is generally satisfactory, but the author's statement that "perhaps the most common cause of failure of root-resorption of the temporary teeth is to be found in septic conditions of their roots," cannot be accepted. While it is true that this condition does interfere with resorption, it is equally true that the permanent teeth are so frequently erupted independently of the *living* deciduous, and that without resorption having taken place in the latter, septic conditions cannot be regarded as an important factor in non-resorption.

The statement that "it is rare that abnormalities are associated with the progress of the first or second permanent molars," requires modification. While true of the first, on account of ample room in the jaw, it is not true of the second molars. These teeth, developed as they are, in the inferior maxillæ, in the rami of the jaw, are necessarily subjected to much of the difficulty of eruption experienced by the third molars. In the attempt to assume the direct vertical position in the jaw, the second molar occupies a very narrow position at the angle, with the liability of impingement upon the inferior dental nerve. This is liable to occur about the eighth or ninth year, resulting in severe nervous disturbances, frequently of an alarming character. This is not generally recognized as it should be. The reviewer has frequently aborted convulsions at this age by a careful watching of symptoms, coupled with deep lancing.

Some criticism seems necessary in regard to the treatment of the pathological conditions following the eruption of the third molars, more particularly in the inferior maxillæ. The author states that "should the mesial half of the crown be free and the posterior half covered by a curtain of gum," it is advised that "the pocket between the tooth and gum be eradicated." This is well, and the subsequent treatment is in agreement with prescribed rules; but the author fails to recognize that the cause of retardation of the third molar, otherwise normally erupting, lies not in the overlapping flap of gum, but that it is obstructed in its advance by the superior third molar. This is the explanation in the majority of cases. Where both of these teeth, superior and inferior, have been erupted synchronously, there should be no irritation, as the jaw is developed *pari passu.* Yet it may occur by pressure of the superior third molar upon the gum. The treatment here is not through sedatives or antiseptics, although both are valuable, but in an intelligent attention to the cause of the inflammation, the malposition of the teeth, which must be corrected either by extraction or excising a portion of the occlusal surface of the enamel upon one or both of the offending teeth.

In the chapter on "Malformations and Malpositions of the Teeth," it is to be regretted that the author fails to give a satisfactory method of diagnosing an impacted third molar, resting horizontally in the jaw and necessarily invisible. It is clearly evident that the third molar, entering the inferior maxilla from the ramus, is liable to this position; and it is further in evidence that if it meets with no obstruction, it will continue its progress forward, and, ordinarily, will not be a cause of reflex disturbance. If, however, *all the teeth* anterior to and including the second molar, are in place, the tooth must stop with impingement upon the roots of the second molar. This means pressure posteriorly upon the inferior dental nerve, and protracted suffering to the patient. It would seem, therefore, a simple thing to state that if *all the teeth* are in place upon that side with the exception of the third molar, with reflex pain present, the cause must be an impacted third molar situated as described. It then becomes a simple matter to find it with the explorer. This is such a serious lesion that it would have been more satisfactory to have had the entire subject fully explained.

It is impossible to agree with the author that "there is no evidence that green deposits stand in causative relation to enamel decalcification." While it is true that green stain upon mature teeth has but a moderate effect upon enamel, it does produce serious destruction upon young teeth. While the author is in accord, in making this statement, with some of the highest authorities, it is not in harmony with the reviewer's experience.

The chapter on "Dental Caries" naturally attracts the dental reader, and he will not be disappointed as to the careful *résumé* of the whole subject. The author devotes space to the more recent investigation of Miller, but fails to do justice in his "History" to the steps that led up to Miller's work. The reviewer would suggest that, in a future second edition, this chapter should be carefully revised. No mention is made of Bell, the advocate of the pure vital theory of dental caries, or of Erdl, the originator of the germ theory, or of Licinus, who has the credit of this, or of Klencke, who, in part, supported it. The work of Leber and Rottenstein is alluded to more briefly than seems to be warranted by its value. The modern idea of bacterial invasion in caries really dates from the work of these distinguished observers, and found its final culmination in that of Miller.

The following quotation indicates that the author is a full adherent to the dictum of Black, that "the hardness or softness of teeth, the amount of calcium salts they contain, or even their anatomical alterations, cannot, as shown by Black, prevent the advent or progress of dental caries, although they undoubtedly do modify its character and rate of progress." If it "cannot prevent" progress, what becomes of the so-called eburnated dentine in which caries has ceased altogether? That the "hardness" of teeth does determine the rate of progress of decay is a well-settled fact in clinical practice, and it is a waste of time and trying to the patience of practitioners to attempt to deny the evidence of experience.

The definition of abrasion, as given, does not meet the conditions that result in the wearing down of teeth. It is ascribed to "the friction of mastication when gritty substances are present." It should be a recognized fact, but unfortunately it is not, that acids play a prominent part in this reduction of the occlusal surfaces of teeth. This has been fully demonstrated, and combined with attrition, with or without gritty materials, there will be a continued loss of substance. The author overlooked the reviewer's work in this direction, in which he demonstrated the acid reaction of the oral fluids in all conditions of rest, and this being especially true at night, when the alkaline salivary secretions are reduced to a minimum. This is not only a prolific cause of the earlier destruction in caries, but also a direct producer of erosion as well as abrasion. Prior to these investigations this fact had not received attention. Kirk subsequently demonstrated the acid action of the mucous follicles of the lip as being an additional factor in the destruction.

The treatment of "Hypersensitivity of Dentine" will generally be regarded as meeting all conditions, but it is not exactly true that "ever since a belief in the chemical nature of dental caries has been accepted, writers upon dental pathology have ascribed the hypersensitivity of dentine in caries to be due to the action of an acid, and have advised the use of alkalies to lessen the sensibility." While the reviewer does not desire to claim special credit, it is due to the history of the subject to say that, up to the time he recommended sodium bicarbonate to lessen sensitivity, there were no agents openly advocated for this purpose except the escharotics, zinc chloride, carbolic acid, and even arsenous oxide.

It is a gratification to find that the author explains the serious objection to the use of caustic alkalies. The extended use of these in the so-called "Robinson remedy" has become a dangerous method of practice, and the practitioners who make use of these and similar protoplasmic devitalizers should take the author's words seriously into consideration.

The reviewer fails to find under "Inflammation of the Dental Pulp, or Pulpitis," any allusion to a recognized condition known and described by writers as superficial pulpitis. This is understood to be an increased vital action in the superficial cells of the pulp caused by continued or slight irritation. In character, but not in extent, it resembles the hypertrophy of the pulp known as fungoid pulp, but must be differentiated from this. The author fails to describe it properly under "Chronic Degenerations of the Pulp." Attention is called to a typographical error in the omission of the umlaut in Arkövy. This is correct in the illustration, but not in the text.

The author writes, "Iodoform, also used as an analgesic ingredient in some prescriptions, is of doubtful value." This is quoted from "Effect of Arsenic on the Pulp." This statement would occasion more surprise were it not for the fact that this is a view generally held. That it is a very mistaken conception of the value of this drug hardly needs assertion, for it is a well-recognized fact that there is no agent in the entire pharmacopœia that possesses the anæsthetic property to a greater degree. In this respect it equals chloroform, with the advantage over this in not being dangerous and retaining a permanent analgesic effect upon the tissue. When properly used in connection with arsenic for devitalization of pulps, it is effectual in reducing the pain and rendering it possible to make an immediate application to an irritated pulp. It must, however, be used properly, and not in the shape of a formula ready prepared.

It is rather surprising that no mention is made of the amount of arsenic to be used upon the pulp of a single-rooted tooth. No warning is given the student of certain possibilities that may happen by the use of an excess of arsenic. The application of this dangerous agent in this way is unscientific, and equally so is the recommendation of "devitalizing fibre." This has no business in the hands of any careful operator, and should long ago have been excluded from practice.

The author quotes Miller with crediting Witzel with the first systematic observations in making the pulp permanently aseptic. He says, "Witzel, in 1874, devitalized the crown portion of the pulp by means of arsenic, extirpated that portion, leaving the pulp in the canals undisturbed, their exposed ends being treated as freshly exposed pulps." It is true that Witzel made this statement, but twelve years later, 1886, he speaks of the shrinking of the pulps in most cases to antiseptic threads. He also, in the mean time, had changed his capping material. The reviewer always understood that Witzel had in view non-infected pulps, or what is generally known as superficial pulpitis. Dr. Allport, of Chicago, years before Witzel, advocated a somewhat similar method of amputation of the pulp.

It is impossible to coincide with the treatment given for pericementitis, for while the reviewer recognizes the absolute necessity of treating the cause, and in this respect is in full accord with the author, it remains true that the methods advised will tend to produce serious complications. It is difficult to understand the author's treatment of alveolar abscess, which follows in natural order. He objects to "hot applications and the use of counter-irritants upon the gum," and prefers "bloodletting by means of a leech." The latter has no place among the counter-irritating agents of the careful operator, for it is not only disgusting but involves the danger of blood-infection.

The chapter on "Pericemental Diseases" includes "Salivary Calculus," which is an excellent presentation of the subject and very perfectly illustrated. This is followed by a dissertation on "Pyorrhœa Alveolaris," with which, it is needless to say, the reviewer does not agree. Considerable space is given to gouty pericementitis, which yet remains to be proved as an existent pathological condition.

The remaining three chapters are on "Diseases of the Deciduous Teeth and their Treatment, Reflex Disorders of Dental Origin, and Infections of and from the Mouth and Sterilization."

Section VII. is upon "Dental Pharmacology and Materia Medica." This will no doubt prove a valuable addition as a matter of convenience to the student for reference.

The reviewer has not thought it necessary to call special attention to the value of each chapter or subchapter, as these exhibit, page by page, the great care taken by the author to condense not only his varied experience, but that of others, in a form best adapted for the needs of the student and active practitioner. It has been deemed the most fitting for one engaged in the same line of teaching to select here and there differences of opinion and practice and to give our readers a glimpse of the leading thought in the volume. This has not been done in the spirit of hypercriticism, but to indicate prominent points in the work and to demonstrate wherein he disagrees with the author, not in a captious spirit, but with a desire, common to all conscientious workers, to reach a common standard of practice. The fact that there exists a wide difference of opinion between the author and reviewer constitutes no objection to the book, for men must differ, and age and experience are no infallible tests and may often take wrong paths. Experience has, however, one merit in that it results in positive opinions, and while the avoidance of dogmatism may be a virtue, its opposite, a too free yielding to a variety of theories, has a tendency to produce confusion in the untrained mind.

Notwithstanding all that has been written by the reviewer, he still regards this work of Dr. Burchard's as the most valuable and practical that has yet appeared upon dental pathology.

Attention has been called to a few things that seem to need modification and correction, but these can all be attended to in the second edition, sure to come in the near future. The book, as it stands, will be accepted, without doubt, by all colleges of dentistry in the United States, for the teachers in this branch have waited impatiently for a text-book to recommend to their several classes. That it will eventually become the standard work on dental pathology and therapeutics can scarcely be questioned, as it is free from much of the objectionable features common to books prepared by a single individual.

The publishers are entitled and should receive the thanks of the entire dental profession for the manner in which this work has been issued, and all those that have preceded it. The efforts they have made to give to dentistry satisfactory works on the various branches taught should be met by a corresponding effort on the part of teachers to increase the circulation among students.

